# Independent and synergistic effects of pain, insomnia, and depression on falls among older adults: a longitudinal study

**DOI:** 10.1186/s12877-020-01887-z

**Published:** 2020-11-23

**Authors:** Yuxiao Li, Minhui Liu, Xiaocao Sun, Tianxue Hou, Siyuan Tang, Sarah L. Szanton

**Affiliations:** 1 Central South University, Xiangya School of Nursing, Changsha, 172 Tongzipo Road of Yuelu District, Changsha, 410013 Hunan China; 2 Johns Hopkins University School of Nursing, Baltimore, MD USA; 3 Department of Health Policy and Management, Johns Hopkins University Bloomberg School of Public Health, Baltimore, MD USA

**Keywords:** Falls, Pain, Insomnia, Depressive symptoms, Older adults

## Abstract

**Background:**

Few studies have examined the relationship between falls and pain, insomnia and depressive symptoms which are common and risk factors in older adults. We aimed to examine the independent and synergistic effects of these risk factors on future falls among older adults.

**Methods:**

We used data of 2558 community-dwelling older adults from 2011 (Y1) to 2015 (Y5) of the National Health and Aging Trends Study (NHATS). Pain was determined by whether participants reported bothersome pain in the last month. Insomnia was assessed by two questions about how often the participants had trouble falling asleep and maintaining sleep. Depressive symptoms were assessed by Patient Health Questionnaire-2. Generalized estimation equation (GEE) models were used to examine the independent effects of pain, insomnia and depressive symptoms at prior-wave (period y-1) on falls at current wave (period y) adjusting for covariates (age, sex, education, race/ethnicity, living arrangement, BMI, smoking, vigorous activities, number of chronic illnesses and hospitalization). The significance of the three-way interaction of these factors (pain*insomnia*depression) was tested using the aforementioned GEE models to determine their synergistic effects on falls.

**Results:**

Overall, the participants were mainly 65–79 years old (68%), female (57%) and non-Hispanic White (70%). At Y1, 50.0% of the participants reported pain, 22.6% reported insomnia and 9.9% reported depressive symptoms. The incidence of falls from Y2 to Y5 was 22.4, 26.0, 28.3, and 28.9%, respectively. Participants with pain (Odds ratio [OR], 95% confidence interval [CI] = 1.36, 1.23–1.50) and depressive symptoms (OR, 95% CI = 1.43, 1.23–1.67) had high rates of falling adjusting for covariates. After further adjustment for insomnia and depressive symptoms, pain independently predicted falls (OR, 95% CI = 1.36, 1.22–1.51). Depressive symptoms also independently predicted falls after further adjusting for pain and insomnia (OR, 95% CI = 1.40, 1.20–1.63). After adjusting for pain and depression, the independent effects of insomnia were not significant. None of the interaction terms of the three risk factors were significant, suggesting an absence of their synergistic effects.

**Conclusions:**

Pain and depressive symptoms independently predict falls, but synergistic effects seem absent. Further research is needed to develop effective strategies for reducing falls in older adults, particularly with pain and depressive symptoms.

## Background

Globally, falls constitute a significant health concern for older adults and are the leading cause of accidental or unintentional injury deaths among the elderly [1]. Falls also lead to disability, hospitalization, loss of independence and psychological problems in older adults [2], resulting in substantial medical and economic costs [3]. According to the World Health Organization (WHO), 30% of older adults over 65 years and 50% of older adults over 80 years report falling at least once a year globally [4]. Identifying risk factors for falls and developing effective intervention strategies are public health priorities in many countries [5].

Pain, insomnia, and depressive symptoms are common health problems in older adults and may contribute to falls [6–9]. More than 50% of US older adults report pain and the majority of them have multiple pain sites [10]. Feet, hips, and knees were the most common sites of pain reported by fallers [8]. Pain contributes to mobility deficits, impaired gait, balance deficits, and muscle weakness, all of which are recognized intrinsic risk factors of falls [11–14]. Older adults with pain are more likely to have fallen in the past year and fall again in the future [7, 12].

The prevalence of insomnia ranges from 21.3 to 34% and it increases with age [15, 16]. Previous studies have shown that insomnia is also associated with a higher risk of falling [8, 17]. Insomnia is related to daytime hypersomnolence, worse physical and cognitive function, difficulty concentrating, and slowed responses, all contributing to increased risk of falling [17]. In addition, the use of sleep medications can increase the risk of falls [18].

Depressive symptoms refer to the presence of unease and somatic complaints that are often accompanied by associated features of depression, but do not necessarily meet the diagnostic criteria for clinical depression [19]. Approximately 15% of older adults report having depressive symptoms [20]. It has been identified as a risk factor for falls in a number of prospective studies [9, 21]. One possible mechanism is depressive symptoms tend to be associated with greater levels of physical impairment and cognitive deficits [22]. Another possible mechanism is that antidepressants can affect attention, gait, balance, and blood pressure regulation [23].

Previous studies concluded that the pain, insomnia and depression are linked through common mechanisms, such as neurobiological and psychosocial mechanisms [24, 25]. Thus, pain, insomnia, and depressive symptoms usually co-occur and may interact to create greater risks of falling than single risk factor among older adults. Patients with both pain and insomnia had worse impairment in physical and psychosocial functioning [26]. Also, co-existence of pain and depression is associated with a greater burden to the individual and society than either symptom alone [27]. When patients have concurrent pain and depressive symptoms, they develop more functional disability [28] and have higher medical costs [29] than those who have either pain or depression. One study suggested that pain and insomnia should be treated together to effectively prevent falls among older adults, as the geriatric syndromes might affect each other [30]. These complex relationships and interactions between pain, insomnia and depressive symptoms are supported the Theory of Unpleasant Symptoms (TOUS) [31]. This theory suggests synergistic effects of interrelated symptoms on functional outcomes, meaning that the cumulative effect of symptoms on outcomes is greater than the sum of the individual symptoms. However, few studies have examined pain, insomnia and depressive symptoms together on fall-related health outcomes. The individual and synergistic effects of pain, insomnia and depressive symptoms on falls in older adults remain unclear.

Thus, the purpose of this study was to examine the independent and synergistic effects of pain, insomnia, and depressive symptoms on future falls among older adults. The synergistic effects were determined by testing the significance of the three-way interaction term of the three factors. We hypothesized that there was a presence of synergistic effects among pain, insomnia, and depressive symptoms contributing to future falls among older adults.

## Methods

### Study design, setting, and sample

We used data from year 2011 (Y1) to year 2015 (Y5) of the National Health Aging Trends Study (NHATS), a nationally representative longitudinal cohort study that has collected a sample of Medicare beneficiaries aged 65 or older in the U.S. Data have been collected annually since 2011 [32]. In Y1 (Year 2011), 8245 interviews were completed, representing an unweighted response rate of 71.3%. We included 7609 participants who were living in community settings. A total of 3624 participants provided data on falls and the prior-year data on pain, insomnia and depressive symptoms from Y1 to Y5. The final analytical sample was 2558 after excluding 1066 participants who reported falls in Y1. Compared to those who were included in this study, the excluded participants were older and had significantly lower education levels, less obesity, less vigorous activities, more chronic conditions, and more hospitalizations.

### Measurements

#### Dependent variable

Fall Participants were asked to report whether they had experienced a fall (yes or no) last year. A fall refers to any fall, slip, or trip in which one loses balances and lands on the floor, ground or at a lower level. Participants were considered fallers if they had any falls in Y2 to Y5.

#### Independent variables

##### Pain

Bothersome pain was defined as moderate to severe pain intensity with low level of interference with life activities [33]. The NHATS interview contained questions about bothersome pain. Participants were asked “In the last month, have you been bothered by pain?” Responses were either yes (coded as 1) or no (coded as 0).

##### Insomnia

Insomnia (including problems with initiating and maintaining sleep) over the past month was assessed using the following two questions: (a) “How often does it take you more than 30 minutes to fall asleep at night?” and (b) “How often do you have trouble falling back to sleep on nights after waking up from sleep?” Response options for these questions included every night, most nights, some nights, rarely, and never. It was coded as insomnia if the participant answered “every night”, “most nights” or “some nights” to either question.

##### Depressive symptoms

Depressive symptoms were assessed using the Patient Health Questionnaire-2 (PHQ-2), which measured how often the participant had been bothered by (a) “little interest or pleasure in doing things” and (b) “feeling down, depressed or hopeless” over the last month. Responses were on a four-point Likert scale, from “not at all” (0), “several days” (1), “more than half the days” (2), to “nearly every day” (3) [34]. We summed scores on both of the PHQ-2 questions to create a score from 0 to 6. Following the recommendation from previous literature, a cut-off of 3 or higher was used to determine depressive symptoms; this cut-off has a sensitivity of 83% and a specificity of 90% for depression [35].

#### Covariates

##### Demographic variables

Demographic characteristics included age (coded 0 for 65–79 years old and coded 1 for 80–90+ years old) [36]; sex (coded 0 for female and coded 1 for male) [37]; education (coded 0 to 3 for “less than high school, high school graduates, some college or vocational school, bachelor or higher”) [36]; race/ethnicity (coded 0 to 3 for “non-Hispanic White, non-Hispanic Black, Hispanic, all other”) [38]; living arrangement (coded 0 to 3 for “alone, with spouse/partner only, with others only, with spouse/partner and with others”) [36].

##### Health-related variables

Health-related covariates included the following: body mass index (BMI) (coded 0 for normal and coded 1 for obesity) [39]; smoking (coded 0 for never smokers and coded 1 for current/former smokers) [40]; whether they ever spent time on vigorous activities in the last month (coded 0 for no and coded 1 for yes) [40]; the number of chronic illnesses (high blood pressure, heart attack/heart disease, arthritis, osteoporosis, diabetes, lung disease, stroke, and cancer) diagnosed by a doctor (coded 0 for no disease, coded 1 for have 1–3 diseases and coded 2 for have more than 4 diseases) [36, 40]; hospitalized in the last 12 months (coded 0 for no hospitalization and coded 1 for at least one hospitalization) [36].

### Statistical analyses

Frequencies and proportions were used to describe demographic and health-related factors. Chi-square tests were used to compare demographics and health-related factors between the fallers and non-fallers in Y1. We then used Chi-square tests to examine the associations between pain, insomnia and depressive symptoms at Y1 and falls in Y2, Y3, Y4 and Y5.

To analyze the longitudinal associations between the three lagged risk factors and falls, we used generalized estimating equations (GEEs) with a binomial distribution to model the prior-wave (period y-1) risk factors on the probability of fall at each current wave (period y) assessment using the log link function. An exchangeable working correlation structure was used in the GEE models. GEE models are an extension of generalized linear models for analyzing longitudinal data. All the measures for the three risk factors and all the covariates are repeated measures, except for several time-invariant variables (e.g., sex, race/ethnicities, educational levels). To examine the synergistic effects of pain, insomnia and depression on falls, we first analyzed by including any two risk factors as independent variables and their two-way interaction term in the GEE model. We then included pain, insomnia and depressive symptoms as independent variables in the model and tested the significance of the three-way interaction term. Independent effects of pain, insomnia and depressive symptoms on falls were examined in three steps. First, we included a single risk factor as the independent variable without adjusting for the other two risk factors. Second, we included a single risk factor as the main predictor and adjusted for another risk factor at a time. Third, we included a single risk factor as the main predictor and adjusted for the other two risk factors. Three models were estimated for each association. Model 1 presented the crude effects with only risk factors of interest included. Model 2 adjusted for demographics (age, sex, education, race/ethnicity, living arrangement) and Model 3 further controlled for health-related variables (BMI, smoking, vigorous activities, number of chronic illnesses, hospitalization). Missing values on fall outcomes and risk factor predictors from Y1 to Y5 were less than 1.5%, and thus no particular technique was used to handle missing data. All analyses were performed using Stata, version 14; *p* < 0.05 (two-tailed) was used to indicate statistical significance. Odds ratios (ORs) and 95% confidence intervals (CIs) were reported from GEE models.

## Results

Overall, about 68% of participants in the sample were 65–79 years old and 57% were female. The majority were non-Hispanic White (70.1%), followed by Black (21.7%). About half of the sample received some college or vocational school education or higher. Significant differences in baseline characteristics were observed between fallers and non-fallers: White and older participants were significantly more likely to experience a fall. Older adults with more chronic illnesses were at higher risk of falling (Table 1).

**Table 1:** Demographic and health-related variables distribution by Y2 fall status, n (%)

Variables	Total (*N* = 2491-2558)	Fall (*n* = 560–572)	No fall (*n* = 1931-1977)	*p*
Age				0.016
65–79	1741 (68.3)	367 (64.2)	1374 (69.5)	
80–90+	808 (31.7)	205 (35.8)	603 (30.5)	
Sex				0.106
Female	1458 (57.2)	344 (60.1)	1114 (56.3)	
Male	1091 (42.8)	228 (39.9)	863 (43.7)	
Education				0.416
Less than high school	567 (22.4)	131 (23.0)	436 (22.2)	
High school graduates	670 (26.5)	155 (27.2)	515 (26.3)	
Some college or vocational school	623 (24.6)	125 (22.0)	498 (25.4)	
Bachelor or higher	670 (26.5)	158 (27.8)	512 (26.1)	
Race				0.007
White, non-Hispanic	1787 (70.1)	432 (75.5)	1355 (68.5)	
Black, non-Hispanic	552 (21.7)	100 (17.5)	452 (22.9)	
Hispanic	126 (4.9)	28 (4.9)	98 (4.9)	
Indian/Asian/Native/Hawaii/Other	84 (3.3)	12 (2.1)	72 (3.6)	
Living				0.934
Alone	825 (32.5)	188 (33.0)	637 (32.3)	
With spouse/partner only	1127 (44.4)	255 (44.8)	872 (44.2)	
With others only	338 (13.3)	72 (12.7)	266 (13.5)	
With spouse/partner and with others	250 (9.8)	54 (9.5)	196 (10.0)	
BMI				0.700
Normal (< 30 kg/m^2^)	1772 (71.1)	402 (71.8)	1370 (70.9)	
Obesity (≥30 kg/m^2^)	719 (28.9)	158 (28.2)	561 (29.1)	
Smoking				0.717
No	2371 (93.0)	534 (93.4)	1837 (92.9)	
Yes	178 (7.0)	38 (6.6)	140 (7.1)	
Vigorous activities				0.155
No	1496 (58.7)	350 (61.3)	1146 (58.0)	
Yes	1052 (41.3)	221 (38.7)	831 (42.0)	
Number of chronic illnesses				< 0.001
0	266 (10.4)	55 (9.6)	211 (10.7)	
1–3	1773 (69.6)	359 (62.8)	1414 (71.5)	
4+	510 (20.0)	158 (27.6)	352 (17.8)	
Hospitalization				0.895
None	2097 (82.3)	472 (82.5)	1625 (82.3)	
At least one hospitalization	450 (17.7)	100 (17.5)	350 (17.7)	

At Y1, half of the participants reported pain (50.0%), 22.6% reported insomnia, and 9.9% reported depression. Across all follow-up years from Y2 to Y5, 22.4–28.9% of the participants reported falls in the last year. During follow-ups, the incidence of falls was 56.5–58.8% for those with pain at Y1, 24.9–27.3% for those with insomnia at Y1, and 12.0–13.6% for those with depression at Y1 (*P* < .05). The effect of pain on the incidence of falls in the older adults during follow-ups was statistically significant, as was depression. However, insomnia was significantly associated with falls only at Y2 and Y4 (Table 2).

**Table 2:** Relationship between baseline (Y1) symptoms and falls during follow-ups (Y2 to Y5), n (%)

	Total	Y2			Y3			Y4			Y5		
		Fall	No fall	*p*	Fall	No fall	*p*	Fall	No fall	*p*	Fall	No fall	*p*
Pain				< 0.001			< 0.001			< 0.001			< 0.001
Yes	1274 (50.0)	331 (57.9)	943 (47.7)		389 (58.8)	890 (47.1)		408 (56.5)	867 (47.4)		418 (56.8)	858 (47.3)	
No	1275 (50.0)	241 (42.1)	1034 (52.3)		273 (41.2)	998 (52.9)		314 (43.5)	961 (52.6)		318 (43.2)	956 (52.7)	
Insomnia				0.097			0.001			0.094			0.001
Yes	574 (22.6)	143 (25.1)	431 (21.8)		181 (27.3)	395 (21.0)		179 (24.9)	397 (21.8)		199 (27.2)	378 (20.9)	
No	1969 (77.4)	426 (74.9)	1543 (78.2)		481 (72.7)	1486 (79.0)		541 (75.1)	1426 (78.2)		533 (72.8)	1433 (79.1)	
Depression				0.013			< 0.001			0.013			0.020
Yes	251 (9.9)	72 (12.6)	179 (9.1)		90 (13.6)	162 (8.6)		88 (12.2)	163 (9.0)		88 (12.0)	163 (9.0)	
No	2286 (90.1)	498 (87.4)	1788 (90.9)		570 (86.4)	1715 (91.4)		631 (87.8)	1655 (91.0)		642 (88.0)	1644 (91.0)	
Total		572 (22.4)	1977 (77.6)		662 (26.0)	1888 (74.0)		722 (28.3)	1828 (71.7)		736 (28.9)	1814 (71.1)	

Table 3 presents the synergistic effects of pain, insomnia and depression on falls. As shown, none of two-way or three-way interaction terms were statistically significant, suggesting an absence of synergistic effects.

**Table 3:** Synergistic effects of lagged pain, insomnia and depressive symptoms on falls

Variables^a^	Model 1OR (95%CI)	Model 2 OR (95%CI)	Model 3 OR (95%CI)
Pain * Insomnia	1.03 (0.83–1.28)	1.01 (0.83–1.29)	0.98 (0.78–1.23)
Pain * Depression	1.04 (0.78–1.39)	1.05 (0.78–1.41)	1.02 (0.76–1.38)
Insomnia * Depression	1.31 (0.98–1.75)	1.30 (0.97–1.74)	1.28 (0.94–1.73)
Pain * Insomnia* Depression	1.32 (0.85–2.06)	1.32 (0.84–2.07)	1.25 (0.78–1.98)

^a^Each two-way or three-way interaction term was tested separately in the models

Model 1: independent variables of interest

Model 2: Model 1 + demographic covariates (age, sex, education, race/ethnicity, living arrangement)

Model 3: Model 2 + health-related covariates (BMI, smoking, vigorous activities, number of chronic illnesses, hospitalization)

Table 4 shows the independent effects of lagged pain, insomnia and depression on falls. After adjusting for demographics and health-related variables, falls were significantly correlated with pain (Odds ratio [OR], 95% confidence interval [CI] = 1.36, 1.23–1.50) and depression (OR, 95% CI = 1.43, 1.23–1.67). Insomnia (OR, 95% CI = 1.15, 1.02–1.29) was associated with falls before adjusting for health-related variables, but after adjustment, insomnia (OR, 95% CI = 1.13, 1.00–1.27) was not significantly associated with falls. In addition, the relationship between insomnia and falls was no longer significant when depression symptoms were in the model.

**Table 4:** Independent effects of lagged pain, insomnia and depressive symptoms on falls

Variables	Model 1OR (95%CI)	Model 2 OR (95%CI)	Model 3 OR (95%CI)
Pain only	1.39 (1.27–1.54) ^***^	1.42 (1.28–1.56) ^***^	1.36 (1.23–1.50) ^***^
Insomnia only	1.16 (1.03–1.30) ^*^	1.15 (1.02–1.29) ^*^	1.13 (1.00–1.27)
Depression only	1.44 (1.25–1.66) ^***^	1.49 (1.29–1.73) ^***^	1.43 (1.23–1.67) ^***^
**Pain & Insomnia**			
Pain	1.39 (1.26–1.53) ^***^	1.41 (1.28–1.56) ^***^	1.36 (1.23–1.51) ^***^
Insomnia	1.13 (1.00 ^§^-1.27) ^*^	1.12 (1.00 ^§^-1.26) ^*^	1.11 (0.98–1.25)
**Pain & Depression**			
Pain	1.40 (1.27–1.54) ^***^	1.42 (1.28–1.56) ^***^	1.36 (1.22–1.50) ^***^
Depression	1.39 (1.21–1.61) ^***^	1.44 (1.25–1.67) ^***^	1.40 (1.21–1.63) ^***^
**Insomnia & Depression**			
Insomnia	1.12 (1.00–1.26)	1.12 (0.99–1.26)	1.09 (0.97–1.23)
Depression	1.42 (1.23–1.64) ^***^	1.47 (1.27–1.71) ^***^	1.43 (1.23–1.66) ^***^
**Pain & Insomnia & Depression**			
Pain	1.39 (1.26–1.54) ^***^	1.41 (1.28–1.56) ^***^	1.36 (1.22–1.51) ^***^
Insomnia	1.09 (0.97–1.23)	1.09 (0.97–1.23)	1.07 (0.95–1.21)
Depression	1.38 (1.20–1.60) ^***^	1.44 (1.24–1.66) ^***^	1.40 (1.20–1.63) ^***^

Model 1: independent variables of interest

Model 2: Model 1 + demographic covariates (age, sex, education, race/ethnicity, living arrangement)

Model 3: Model 2 + health-related covariates (BMI, smoking, vigorous activities, number of chronic illnesses, hospitalization)

*p < 0.05; ***p < 0.001

^§^ Please note that this figure looks like the confident interval includes 1. However, the *p*-value was still < 0.05

When both pain and insomnia were incorporated into Model 1 and all the covariates were adjusted, pain (OR, 95% CI = 1.36, 1.23–1.51) was significantly associated with falls; insomnia (OR, 95% CI = 1.11, 0.98–1.25) was not significantly associated with falls. When pain and depression were both included in the equation, pain was significantly associated with falls after adjusting for all the covariates (OR, 95% CI = 1.36, 1.22–1.50), as was depression (OR, 95% CI = 1.40, 1.21–1.63). Both insomnia and depression were included in Model 1. After adjusting for all the covariates, insomnia was not significantly associated with falls (OR, 95% CI = 1.09, 0.97–1.23); depression was significantly associated with falls (OR, 95% CI = 1.43, 1.23–1.66). When pain, insomnia and depression were all incorporated into the equation and adjusted for all covariates, pain (OR, 95% CI = 1.36, 1.22–1.51) and depression (OR, 95% CI = 1.40, 1.20–1.63) were significantly associated with falls; insomnia was not significantly associated with falls (OR, 95% CI = 1.07, 0.95–1.21).

## Discussion

To our knowledge, this study is the first attempt to investigate the independent and synergistic effects of pain, insomnia, and depressive symptoms on subsequent falls in community-dwelling older adults. The study results suggested that pain and depressive symptoms were independent predictors of falls in community-dwelling older adults; insomnia was not. In addition, the interaction terms of pain, insomnia and depressive symptoms were not significant, suggesting that the effects of pain, insomnia and depressive symptoms on falls are additive.

Contrary to our hypothesis, no synergistic effect of pain, insomnia and depressive symptoms were observed in the present study. The relationship between pain, insomnia and depressive symptoms is complex. Previous studies have suggested a bidirectional relationship between two of the three risk factors [41, 42], or that one risk factor regulates the relationship between the other two [43]. Previous studies have reported a vicious cycle between pain, insomnia, and depressive symptoms, which can make poor outcomes worse, suggesting synergistic effects of these risk factors [44, 45]. However, our study findings did not support the synergistic effects, meaning that effects of these risk factors on falls are additive. Two other studies also found an absence of synergistic effects of pain, insomnia, and depression on healthcare use among older adults with osteoarthritis [46, 47].

Our study found that pain and depressive symptoms were independent predictors of falls after adjusting for insomnia and covariates. This result is consistent with the finding of a previous cohort study that chronic pain and depressive symptoms are independently associated with the risk of falls [48]. The main difference between our study and the previous study is that the prior one did not adjust for insomnia. Thus, our study provided stronger evidence that pain and depressive symptoms may independently lead to falls. Studies have reported that old adults with depressive symptoms had a significantly increased risk of falls [49, 50]. A meta-analysis of prospective studies indicated that any pain in any location is associated with an increased risk of falling [12]. Pain is associated with decreased muscle strength, impaired balance and thus with an increased risk of falls [11]. Our study found that half of the older adults experienced pain. Pain is a remediable risk factor for falls but is not taken seriously. Older adults regard pain as a natural part of aging, and health care professionals often ignore it [51]. Similarly, depressive symptoms are also often overlooked [52]. Both pain and depressive symptoms are common in older adults and are potentially modifiable risk factors for falls. Therefore, effective pain and depression management strategies should be integrated into multifactor fall prevention programs.

In our study, the association between insomnia and falls did not remain significant after adjusting health-related variables. Also, insomnia was not associated with falls when controlling for depressive symptoms. In the previous literature, most studies have found that insomnia was significantly associated with higher risk of falls in older adults [53, 54]. Only one study has reported that there is no association between insomnia and falls [55], but this study included only older women. One explanation for different results could be the different measures of insomnia and confounders selection. Adjustment for other variables suggests that insomnia may be a secondary risk factor of one or more health-related variables. In the model falls on insomnia and depressive symptoms, the association between insomnia and falls disappeared. One previous study has reported similar findings that the presence of depressive symptoms may mediate the association between insomnia and falls among older adults [56]. It also suggests that falls are affected by a variety of factors. Further research is needed to explore what health-related factors influence the relationship between insomnia and falls.

Several limitations should be noted. All measures were retrospectively self-reported and thus subject to recall bias and reporting errors. In particular, a one-year re-call time window of falls might be long for older adults. They may only recall injurious falls. In addition, pain was assessed by a single question and insomnia was evaluated by two questions on two commonly seen sleep problems. Although these questions are not components of validated questionnaires, previous NHATS studies using these two variables provided evidence of criterion and face validity [7, 57, 58].. This study did not adjust for some other fall-related covariates as they were unavailable from the data. Understandably, secondary database data were not collected to specifically determine the risk of falls. Thus, we included a number of health and functional indicators as covariables to adjust for the relationship between independent variables and falls.

## Conclusions

Pain and depressive symptoms independently predict future falls among older adults, but synergistic effects seem absent. Healthcare providers should routinely evaluate and intervene in both conditions to reduce their respective effects on falls. Further research is needed to develop effective strategies for reducing fall risk and related injuries in older adults, particularly in those with pain and depressive symptoms.

## Data Availability

The data sets analyzed in the current study are publicly available: NHATS (https://www.nhats.org/).

## Abbreviations

PHQ-2: Patient Health Questionnaire-2
GEEs: generalized estimating eqs.
BMI: body mass index
NHATS: National Health and Aging Trends Study
U.S.: United States
